# Toward an Embodied Medicine: A Portable Device with Programmable Interoceptive Stimulation for Heart Rate Variability Enhancement

**DOI:** 10.3390/s18082469

**Published:** 2018-07-30

**Authors:** Daniele Di Lernia, Pietro Cipresso, Elisa Pedroli, Giuseppe Riva

**Affiliations:** 1 Department of Psychology, Università Cattolica del Sacro Cuore, Largo Gemelli, 1, 20100 Milan, Italy; p.cipresso@auxologico.it (P.C.); giuseppe.riva@unicatt.it (G.R.); 2 Applied Technology for Neuro-Psychology Lab, IRCCS Istituto Auxologico Italiano, Via Magnasco, 2, 20149 Milan, Italy; e.pedroli@auxologico.it

**Keywords:** interoception, interoceptive stimulation, heart rate variability, C-tactile, affective touch, CT afferents, rehabilitative medicine

## Abstract

In this paper, we describe and test a new portable device that is able to deliver tactile interoceptive stimulation. The device works by delivering precise interoceptive parasympathetic stimuli to C-tactile afferents connected to the lamina I spinothalamocortical system. In humans, interoceptive stimulation can be used to enhance heart rate variability (HRV). To test the effectiveness of the device in enhancing HRV, 13 subjects were randomly assigned in a single-blind between-subjects design either to the experimental condition or to the control condition. In the experimental condition, subjects received stimulation with the developed device; in the control condition subjects received stimulation with static non-interoceptive pressure. Subjects’ electrocardiograms (ECG) were recorded, with sampling at 1000 Hz for 5 min as a baseline, and then during the stimulations (11 min). Time domain analyses were performed to estimate the short-term vagally mediated component (rMSSD) of HRV. Results indicated that the experimental group showed enhanced rMSSD, compared to the control group. Moreover, frequency domain analyses indicated that high frequency band power, which reflects parasympathetic activity in humans, also appeared to be enhanced in the experimental group compared to control subjects. Conclusions and future challenges for an embodied perspective of rehabilitative medicine are discussed.

## 1. Introduction

Traditional approaches to body perception are usually focused upon the role of proprioceptive signals; nonetheless, recent perspectives have identified inputs that come from inside the body (i.e., interoceptive) as core elements in human well-being. This new perspective further defines the concept of “embodied medicine”—a specific approach that considers the fundamental role of the body in all the processes connected to human health [1,2].

From this point of view, interoception represents an emerging and promising topic in neuroscience. Interoceptive perceptions can be defined as the sense of the physiological status of the entire organism [3] and they encompass a broad range of relevant biological functions that serve conscious and unconscious processes. The central component of the interoceptive system is the anterior insular cortex, which receives information through a vast network of small un-myelinated fibers connected to the Lamina I spinothalamocortical pathway. These specific fibers, called C-fibers, compose a poly-modal afferent system that innervates the entire organism and report a wide range of inputs such as: hunger, thirst, pain, itch, temperature [4], muscle contraction [5,6], hormonal and immune activity, and cardiorespiratory function [3,7], along with a specific type of tactile perception called C-tactile (CT) [8]. These inputs are processed in the interoceptive matrix that creates a metarepresentation of the active processes in the organism based on an explicit lateralization of the cortex. Specifically, the left and the right insula are usually coactive in the interoceptive system; nonetheless, parasympathetic inputs are preferentially processed by the left insula [9] while sympathetic ones are usually processed by the right one [3,10].

Recent evidence identified altered interoceptive processes in a broad range of clinical conditions such as chronic pain [11], eating disorders [12,13,14,15], anxiety [16,17], depression [18,19,20,21,22], addictions [23,24], post-traumatic stress disorder [25], insomnia [26], and several others [27,28]. However, a primary limitation in the study of the interoceptive system is the ontological difficulty in accessing and reproducing specific interoceptive stimuli. Although it is quite easy to activate pain and temperature inputs, other interoceptive stimuli (e.g., hunger, thirst, visceral sensations) are quite difficult to reproduce in a controlled manner, reducing the possibility of consistently exploring different aspects of the interoceptive system in controlled settings. Moreover, easily reproducible interoceptive inputs, such as pain and temperature, are generally processed by the right insula [29,30] due to their sympathetic high valence, leaving the whole system of parasympathetic inputs vastly unexplored.

Parasympathetic interoceptive inputs have been recently discovered as a promising research field, and among these kinds of stimuli, CT (or affective touch) is arguably the most interesting one. CT afferent fibers constitute a secondary touch system with a deep involvement in different psycho-physiological pathways [31]. A prominent framework theorized their fundamental role in social contact and emotional bonding [32]. Moreover, several clinical conditions demonstrated alterations in perceptions that were connected to the CT afferents, suggesting an implication for interoceptive touch also in psychopathological functioning [33]. As a further matter, human models indicated that interoceptive touch can reduce and modulate pain perception [34,35,36], while animal models suggested that CT afferents can also modulate anxiety and chronic stress [37,38,39].

This evidence suggests a promising role of CT interoceptive stimulation in different fields, from clinical applications to sensing technology and assessment; nevertheless, the same evidence underlines the need for technological devices that are able to deliver interoceptive tactile stimulation, both for applied and basic research. As a matter of fact, several experimental devices [40,41,42,43] actually exist and are able to deliver interoceptive C-tactile stimulations. Nonetheless, these instruments have several limitations. Specifically, they are usually unable to deliver continuous stimulation, and it is not possible to modify the frequency and the variance of the stimuli. Additionally, these devices are usually connected to a fixed setup, limiting the portability of the instrument in different settings (i.e., hospitals, laboratories) and the possibility to apply stimulation to different body parts of a subject.

To address these issues, the paper describes a new portable device that is specifically designed to deliver tactile interoceptive stimuli. The instrument allows continuous stimulation to any chosen body site and it also allows programming of the variance and the frequency of the delivered stimuli. The device can be used for several purposes; in a recent study [44], interoceptive tactile stimulation delivered by the prototype has been used to behaviorally sense the amount of interoceptive processing in healthy subjects. Results showed that subjects’ ability to correctly evaluate interoceptive tactile stimuli is connected to several pathological conditions, suggesting that interoceptive tactile inputs can be used to sense deficits and alterations on a sub-clinical level, even before a pathological condition is manifested.

In the present study we tested the prototype, collecting ECG data in a single-blind between-subjects study to verify the physiological effects of interoceptive stimulation on healthy subjects.

To validate the effect of the device, we considered evidence from previous literature that indicated a positive effect of interoceptive tactile stimulation upon heart rate variability (HRV) [45]. HRV is a functional time parameter that expresses the variation in time intervals between heartbeats, and it is deeply connected to several health related domains, whereas diminished HRV has been related to a broad range of conditions, from mortality after a myocardial infarction [46] to depression [47].

Specifically, a recent study from Triscoli, Croy [45] reported that interoceptive tactile stimulation can enhance HRV in healthy subjects, compared to non-interoceptive tactile stimulation. Following these results, we presented and validated the interoceptive stimulator in a single-blind between-subjects design, hypothesizing that the device will enhance HRV in the experimental group, compared to a control group that will receive non-interoceptive tactile stimulation (i.e., static pressure). The study will collect ECG data, and HRV will be measured through the rMSSD index, which is a HRV time domain index primarily connected to vagally-mediated changes [48].

## 2. Methods

### 2.1. Technical Development

The interoceptive stimulator has been designed and developed to provide continuous C-tactile stimuli with a programmable pattern of stimulation. To pursuit this goal, the device was been designed with consideration for all the relevant factors connected to CT afferent fibers.

**Figure 1:**
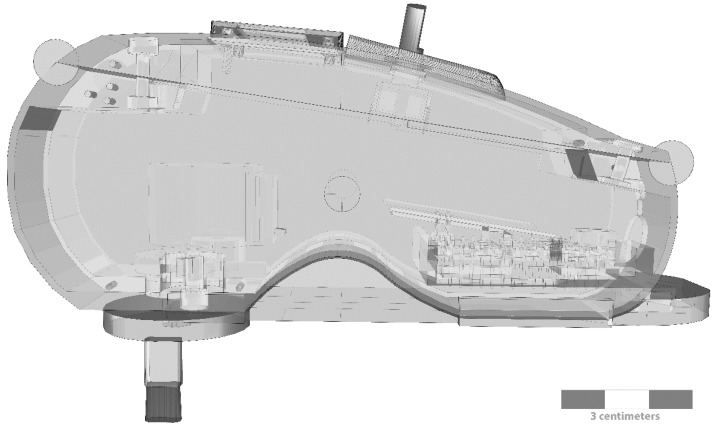
Interoceptive portable stimulator with a 3D-printed case and calibrated probe mounted.

CT fibers constitute a secondary touch system with peculiar characteristics. They are uniquely found in non-glabrous skin where they distinctively respond to light touch with a force under 2.5 mN [49,50] and a stroking velocity between 1 and 10 cm/s [41,51], with a mean peak of activation at around 3 cm/s. Moreover, they exhibit a tendency to fatigue [8,52], and a specific after-discharge pattern with a delayed acceleration effect [49,53]. Considering these factors, the device (Figure 1) uses a step motor, a driver, and an ARDUINO NANO as the main programmable controller to deliver targeted interoceptive C-tactile stimuli.

**Figure 2:**
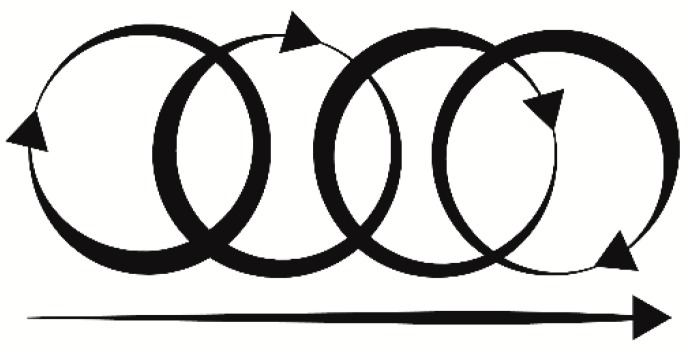
Stimulation pattern with circular and linear components.

A liquid crystal display (LCD) and a digital encoder allow the selection of pre-programmed stimulation patterns directly on the device. A rechargeable battery connected to a direct current (DC) boost ensured different lines to power the step motor (12 V) and the main electronics (3–5 V). The device was enclosed in a specifically 3D-printed case that allowed portability and maneuverability. A specifically designed and calibrated probe was attached to the step motor main shaft, providing tactile stimulation. The probe moved in a circular pattern, with a linear component handled by the operator. By mixing circular and linear stimulation (Figure 2), the probe matched the maximal mean firing frequency of CT afferents (3 cm/s ± 0.5 cm/s) [40], allowing continuous stimulation with a specific pre-calibrated force <2.5 mN that is the optimal threshold for interoceptive touch [49,54,55]. Lastly, the probe had an oval shaped area that matched the receptive CT human afferent patch (≈35 mm^2^) [36,52] ensuring a targeted interoceptive stimulus.

Besides, C-tactile afferents showed a specific behavior of fatigue and in-excitability [8,52] reducing their firing rate to 0 after 5 s of continuous stimulation [36]; therefore, the device factored in several patterns of stimulation for optimal continuous performance. Considering linear and circular stimulation, probe dimension, optimal velocity, and angular motion, the device stimulated a single CT afferent patch for only 0.28 s within a single revolution, allowing for continuous application without inhibition of the receptive CT field.

The device was programmed with ARDUINO IDE native language. It used common open source libraries for the digital encoder and the LCD screen. A custom library for the stepper motor driver was developed to reduce motor time activation; the shaft could therefore reach a specific angular velocity within only 10 ms, providing almost instantaneous interoceptive stimulation within the optimal CT firing range.

The code was updatable via universal serial bus (USB) and specific stimulation patterns could be uploaded into the memory of the device, and selected through the digital encoder and the LCD screen. The device stored stimulation patterns as STRING variables in the PROGMEM, allowing the microcontroller to maintain memory even without power. A set of parameters could be controlled, such as: velocity, duration of stimulation, and pattern of the stimuli. These parameters could be mixed together, creating different types of interoceptive parasympathetic stimulations with various purposes.

**Figure 3:**
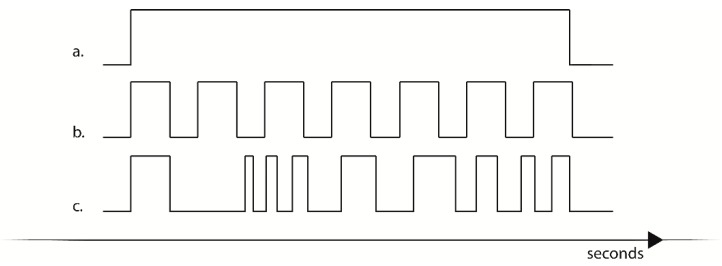
Examples of programmable stimulation. (**a**) Continuous stimulation, (**b**) low variance stimulation, (**c**) high variance stimulation.

In continuous mode (Figure 3a), the device activated a continuous interoceptive stimulus. The duration of the stimulation could be programmed and stored in flash memory; otherwise the device could be stopped at the appropriate time via a coded command selected through the digital encoder.

In variance stimulation (Figure 3b,c) a predetermined pattern of stimuli with defined optimal velocity and duration was programmed in the device and selected through the digital encoder. If selected, the device activated a series of stimuli in a fixed sequence that could be customized either for the duration of a single stimulus, for the duration of the pause between stimuli, or both. Sequences could be therefore either be programmed to deliver a low variance stimulation (Figure 3b) where the device presented interoceptive stimuli with a predictable pattern, or the device could be programmed to deliver high variance stimulation, presenting a pattern of stimulation with a low predictability. Possible applications of this kind of stimulation will be presented in the discussion section.

Lastly, the device implemented a serial port interface (SPI) that allowed synchronization of stimuli between clone interoceptive stimulators in a MASTER-INDEPENDENT SLAVES configuration for up to 256 devices, or in a DAISY CHAIN configuration for a larger number of cloned devices.

### 2.2. Participants

As a part of an on-going research project, a subsample of 13 subjects were recruited through consecutive sampling (nine females and four males; age mean = 36.15 years, SD = 17.59; body mass index (BMI) mean = 22.25, SD = 1.92). The sample size was comparable to previous literature studies with similar variables [56]. Exclusion criteria were the presence of current psychological or physical diagnoses, alterations in tactile perception (paraesthesia), allodynia, and heart-related conditions. Subjects were asked to avoid pharmacological medications during the 12 h prior to the experiment, and nicotine and caffeine in the 2 h prior to the experiment. All subjects gave written informed consent in accordance with the Declaration of Helsinki (2008). The protocol was approved by the Ethics Committee of Catholic University of Sacred Heart of Milan.

### 2.3. Procedure

On arrival, subjects received information about the experiment and gave written consent. The study design was a single-blind between-subjects procedure; participants were informed that the experiment was about time perception. Specifically, they were informed that they were about to receive a series of non-painful tactile stimuli, and that their task was to mentally estimate the time duration of these stimuli. For the single blind procedure, subjects were asked to verbally report the duration of the tactile stimulation (in seconds).

Before the experimental procedure, subjects took part in a brief anamnestic interview with a psychologist specialized in psychopathological assessment. After that, subjects were seated comfortably in a quiet room and they were connected to a BioSignalPlux Bluetooth ECG device (Plux, Lisbon, Portugal) with Ag/AgCl electrode sampling at 1000 Hz. ECG was recorded for the entire duration of the experiment and digitally marked to identify the different phases of the session. A 5 min resting baseline was recorded prior to the stimulation procedure. The tactile stimulation procedure lasted approximately 11 min.

### 2.4. Experimental Design

The study was defined as a single-blind, between-subjects design. Participants were randomly assigned to the control (SHAM) condition or to the experimental (EXP) condition. Both conditions proposed a tactile stimulation and asked the subjects to estimate and verbally report on the duration (expressed in seconds) of the stimuli.

In the EXP condition, subjects received interoceptive tactile (CT) stimulation to the left volar forearm; stimulation was delivered with the developed device. This specific body site was chosen with consideration of microneurographic evidence in the literature that indicated the presence of CT afferents in the volar forearm of healthy subjects [40,49,50,53]. Stimulation of CT afferents was preferentially processed by the left insula [9], as discussed in the introduction.

In the EXP condition, subjects received low variance interoceptive tactile stimulation. Tactile stimulation was delivered in fixed time durations that were randomly proposed in six blocks. Each block was composed of six stimuli of respectively 8 s, 10 s, 12 s, 14 s, 16 s, and 18 s. Each stimulus was followed by a 6 s pause. The entire duration of the stimulation was approximately 11 min.

In the SHAM condition, subjects received a static (100 mN) pressure stimulation to the same body site as the EXP condition (i.e., left volar forearm). The SHAM condition was designed to be tactile non-painful pressure stimulation due to the fact that the pressure activated different tactile receptors that were not preferentially processed by the interoceptive system, but are equally distributed in the volar forearm [40,50]. Static pressure stimuli are preferentially processed by the somatosensory cortex trough Aβ fibers, and therefore they provided an optimal control condition [57]. In the SHAM condition, the tactile pressure stimulation was performed with a plastic cylinder with smooth edges.

In the SHAM condition, participants received a non-interoceptive low variance tactile stimulation (fixed pressure of 100 mN) with the exact same block modalities and stimuli duration as the EXP condition. There was no difference in stimulation duration between the control and the experimental condition.

### 2.5. Time and Frequency Domain Measurements of Heart Rate Variability

HRV is the variation in the time intervals between heartbeats. This variation can be analyzed through different time and frequency domains, providing several indexes that reflect different processes connected to the autonomic nervous system (for an overview see Shaffer and Ginsberg [48]).

In the time domain, the most reliable indexes for long and short time frames are the SDNN index, i.e. the standard deviation of normalized inter-beat interval (NN) intervals, and the rMSSD index, i.e. the root mean square of successive inter-beat interval (RR) intervals [48]. Sympathetic and parasympathetic activities both contribute to SDNN, while rMSSD is primarily connected to vagally-mediated changes [58].

As discussed in the introduction, considering the nature and the effects of interoceptive touch, the study will analyze the rMSSD index to verify whether the stimulation delivered by the prototype is able to enhance HRV, as previous evidence in the literature have demonstrated [45].

Regarding the frequency domain, analyses are segmented into three spectral bands: the very low frequency band (VLF) is between 0.0033 and 0.04 Hz, the low frequency band (LF) is between 0.04 and 0.15 Hz, and the high frequency band (HF) is between 0.15 and 0.40 Hz. Among these bands, HF directly reflects parasympathetic activity, while the LF band is primarily connected to sympathetic activity [48].

ECG recordings were digitally marked at the beginning and at the end of the baseline (5 min). Extracted records between the two digital marks were utilized for the baseline analyses.

ECG recordings for the stimulation procedure were digitally marked at the beginning and at the end of the tactile stimulation. Recordings were extracted for the stimulation procedure, both in the SHAM and EXP condition. For both conditions, a time window of approximately 11 min and 24 s was analyzed for each subject.

HRV analyses were run through BioSignalsPlux propriety software OpenSignals (build 2018-02-27, version 1.0) with a HRV analysis pack, following established guidelines [59]. ECG recordings were manually inspected for ectopic beats, arrhythmic events, missing data, and noise effects. Moreover, according to the Standards of Measurement [59], inter-beat-intervals at greater or less than 20% of the mean of the previous 20 intervals were removed from the analysis to decrease erroneous estimations.

RR intervals were computed by means of QRS complex and peak detection, based on the Pan & Tompkins algorithm. The power spectral density (PSD) was estimated using Welch’s method with a Hanning window of the length of the number of NNs. The data was divided into overlapping segments, and a periodogram was obtained for each segment, enabling the computation of averages and the extraction of spectral features. Standard frequency bands (VLF, LF, HF) were used for the peak, absolute, and relative power computations.

### 2.6. Statistical Analyses

Non-parametric tests were performed to verify that there were no differences between the control and experimental groups at baseline, on both demographic and HRV variables. Although assumptions for parametric tests were satisfied, sample dimension suggested that non-parametric tests could provide more reliable results [60]. To verify the hypothesis that interoceptive stimulation delivered by the prototype enhanced HRV—as represented by the short term component rMSSD—a Mann Whitney U test with Monte Carlo simulation was run for rMSSD as a dependent variable with a factor group (experimental and control).

Due to differences in HF power (ms^2^) between experimental and control groups at post-baseline measures, a similar Mann Whitney U test was run with HF power as a dependent variable, to verify whether the stimulation delivered by the prototype was also able to increase the absolute power in the HF domain, indicating increased parasympathetic activation.

Consequently, a similar Mann Whitney U test was conducted for the LF band power and other HRV variables, to verify that the device did not have effects on the sympathetic activation.

Statistical analyses were performed with SPSS for Windows, version 22.0 (SPSS Inc., Chicago, IL, USA).

## 3. Results

Total sample of N = 13 showed HRV baseline values that were comparable to previous literature evidence [61]. Results are summarized in Table 1. No differences were found between the experimental and control groups at baseline for the main interest variables (Table 2).

Mann-Whitney U Tests with Monte Carlo simulation [seed = 624387341] were performed for variables of main interest. We used one-tailed tests for one-sided hypotheses, considering that we had a priori expectations, namely that rMSSD and HF power would be higher in the EXP condition than in the SHAM condition, due to the enhancing effect of the interoceptive stimulator. According to the guidelines, we reported the confidence interval for the P-value along with the estimated *p*-value [62].

A Mann-Whitney U Test was conducted to determine a statistically significant difference between the group (experimental and control) on rMSSD values during the stimulation. Results indicated that rMSSD was significantly higher (U = 8.500, estimated *p* = 0.036 | 99% confidence interval for *p*: 0.031–0.040) in the experimental group (mean = 51.57; SD = 14.44) than in the control group (mean = 36.83; SD = 8.88). Furthermore, results indicated a significantly enhanced HF power (U = 8.000, estimated *p* = 0.037 | 99% confidence interval for *p*: 0.032–0.042) in the experimental group (mean = 1164.86; SD = 602.69) compared to the control group (mean = 536.17; SD = 527.43). Lastly, results indicated that there was no difference in LF power between the experimental group and the control group (U = 16.000, estimated *p* = 0.274 | 99% confidence interval for *p*: 0.263–0.286). Mann-Whitney U Tests indicated no significant difference in the other main interest variables. Results are summarized in Table 2.

## 4. Discussion

In the paper, we have presented a new portable device that is able to deliver programmable interoceptive tactile stimulation. We tested the device in a single-blind study, collecting ECG data and analyzing HRV in healthy subjects. Analyses focused on rMSSD, a time domain parameter that measures short-term variation in heart rate, primarily reflecting vagally mediated changes [48]. As hypothesized, the stimulation delivered by the prototype effectively enhanced the rMSSD component in the experimental group compared to the control group. These results confirm previous literature evidence regarding the effect of interoceptive touch on HRV [45]. Moreover, analyses showed enhanced HF power in the experimental group, suggesting an effect of the device on the subjects’ parasympathetic system. The results also indicated that the device did not affect the LF band power, suggesting that the prototype was able to provide a selective pattern of activation that was exclusively targeted the parasympathetic system, as was expected due to the nature of CT afferents.

This selective pattern of parasympathetic activation might also explain non-significant effects upon other main variables such as SDNN, LF, and VLF. Specifically, the SDNN component is largely determined by both sympathetic and parasympathetic activity, whereas evidence in the literature also indicated that SDNN is predominantly affected by the LF and VLF components when the relative power of these bands is greater than HF power [48], as in our stimulation condition.

Moreover, non-significant differences in the LF band power indicated that the experimental device was selectively able to stimulate the parasympathetic system without concomitant activation of the sympathetic branches, and this conclusion is also partially supported by the VLF results. Although physiological mechanisms connected to VLF band are not entirely understood, literature has suggested that the VLF band is primarily influenced by the sympathetic activity that is connected to the heart’s intrinsic nervous system [63], thus also excluding an effect of the device upon this specific sympathetic branch. Furthermore, the absence of significant differences in AVG_IHR between experimental and control group, suggested that the effect of the device should not be attributed to a variation in the heart activity because it was not directly mediated by changes in heart rate.

Considering the above results, we can argue that targeted CT parasympathetic stimulation should affect neither the LF band nor the VLF band, with a consequently limited impact upon the SDNN component on short time frames. Nevertheless, evidence suggested that CT stimulations—longer than the one proposed in this study—can also directly influence the SDNN index [45], probably due to a relative shift in the frequency bands that constitute this HRV component, redistributing the mean power in favor of the HF band with a consequent reduction in the LF and VLF components.

Lastly, results also confirmed that the control condition (i.e., static pressure) did not induce any kind of physiological activation, endorsing the usefulness of the selected control procedure and the effects of the device upon the parasympathetic system.

Interoceptive parasympathetic (CT) stimulation has a wide range of applications. The developed device has been recently tested as an assessment instrument [44], whereas parasympathetic activation was able to sense for distortions in interoceptive balance connected to depressive and body distortion disorders in subclinical subjects.

Moreover, parasympathetic interoceptive stimulation has been proven to modulate pain [34,36], anxiety [36], and body ownership [41]; therefore, the device may show applicability for conditions that require intervention both on the clinical [33] and the subclinical levels.

Lastly, the device allows complete control of several key variables. It can be therefore programmed to deliver low or high variance stimulation with specified learning rate curves. Different variance stimulation with programmed learning rate curves has been implemented as a method for promoting neuroplasticity in several applications [64]. The device might therefore employ the same rationale to promote or suppress neuroplasticity in the interoceptive matrix. This kind of application can be theoretically applied to a variety of clinical conditions. Specifically, chronic pain [11], addictions, post-traumatic stress disorder, and insomnia presented hyper-activation in the cortical areas that was linked to the interoceptive matrix [25,26,28,29,30,65]; therefore a low variance stimulation was able to reduce neuroplasticity, and might reduce the processing of sympathetic high arousal interoceptive stimuli in the right insula, improving clinical conditions and decreasing severity of symptoms.

Conversely, a high variance stimulation aimed at enhancing interoceptive neuroplasticity can provide applications for those conditions that are characterized by a low processing of bodily sensations and a functional and structural reduction of the interoceptive cortical areas, such as depression [18,19,20,21], anorexia nervosa, and other eating-related disorders [12,13,14].

Lastly, the device can also be used in a complementary manner along with other technologies such as virtual reality (VR) [66], sonoception [1], and “positive technologies” [67] on a general level [68]. For example, it can be used to improve embodiment and body ownership [41] in VR environments for clinical [69] and assessing purposes [70], to modulate specific interoceptive patterns for treatments [66], or to provide interoceptive stimulation during a variety of other contexts as well (i.e., exposure therapy). These examples summarize some of the possibilities of the interoceptive stimulator; nonetheless, promising evidence in the field of interoception suggests that other practical applications might be developed in the future.

## 5. Limitations

Several limitations impaired the study and the interpretation of the results. Although statistical power and sample assumptions were adequate to the analyses performed, sample size was generally limited. In the manuscript, we addressed this specific limitation by performing additional non-parametric analyses with a Monte Carlo simulation, which allowed us to compensate for the limited sample size. Non-parametric analyses further confirmed the results from the parametric tests; nevertheless, more data will be needed in future studies to coherently explore the field of interoceptive tactile stimulation.

Furthermore, although the main hypothesis of the study was focused upon rMSSD index of HRV, secondary findings identified a significant effect of interoceptive tactile stimulation upon the HF band of HRV, which is an index connected to parasympathetic activation. These results are in keeping with the rationale of the study because interoceptive tactile stimulation is preferentially processed as parasympathetic input by the left insula [9]; however, evidence in the literature has suggested that the HF band can be easily affected by respiration, which was not controlled in the present study.

A specific phenomenon known as respiratory sinus arrhythmia (RSA) was proven to partially modulate parasympathetic heart-related activity. However, literature evidence indicated that the effect of RSA upon the vagal and the parasympathetic activity is indeed quite limited when considering the relation of vagal tone, respiration, and RSA across (between) subjects with normal resting respiration. As Grossman and Kollai [71] reported, “resting RSA does not accurately predict individual differences in cardiac vagal tone”, and “the relationship between individual variations in RSA and vagal tone is not improved by controlling respiratory parameters”. Several other studies supported these conclusions, indicating that respiratory parameters did not influence tonic variations in heart period [72,73,74], especially in inter-individual conditions with healthy resting subjects with normal respiration [75]. Conversely, evidence from literature suggested that respiratory parameters must be controlled when RSA amplitude is used as an index of vagal activity, as a replacement for other direct cardiac measures [72], which was not the design of the current study.

In conclusion, although literature evidence indicated a minimal effect on respiration, we cannot exclude a small interference by respiration upon the HF band; therefore, the result of the vagal tone in healthy resting subjects supports the results of the current study. Nonetheless, the HF power domain should be considered with caution, until future studies collect additional data including respiratory parameters.

## Figures and Tables

**Table 1:** Sample characteristics and heart rate variability (HRV) baseline values.

	N	Min	Max	Mean	SD
Age	13	23	71	36.15	17.59
BMI	13	19.81	25.91	22.26	1.92
SDNN	13	28	69	49.54	11.20
rMSSD	13	12	69	40.46	14.39
AVG_IHR	13	66	91	74.23	6.82
VLF	13	140	2207	901.23	570.32
LF	13	86	3624	881.77	1035.95
HF	13	33	3436	726.46	904.83

BMI: body mass index, SDNN: Standard deviation of all NN intervals, rMSSD: square root of the mean of the sum of the squares of differences between adjacent NN intervals, AVG_IHR: average of the instantaneous heart rate (beats per minute), VLF very low frequency power in ms^2^, LF: low frequency power in ms^2^, HF: high frequency power in ms^2^.

**Table 2:** Results for the experimental (EXP) and control groups (SHAM).

	Baseline					Stimulation				
	EXP (N = 7)		SHAM (N = 6)		*p*	EXP (N = 7)		SHAM (N = 6)		*p*
	Mean	SD	Mean	SD		Mean	SD	Mean	SD	
Age	35.57	18.1	36.83	18.6	0.904	<<	<<	<<	<<	<<
BMI	22.58	2.35	21.88	1.37	0.830	<<	<<	<<	<<	<<
SDNN	48.29	11.10	51.00	12.18	0.774	57.00	12.98	50.50	10.65	0.352
rMSSD	41.00	16.94	39.83	12.31	0.886	51.57	14.44	36.83	8.88	0.036 *
AVG_IHR	75.29	4.19	73.00	9.34	0.132	68.86	11.64	73.17	6.97	0.886
VLF	851.00	409.98	959.83	755.83	0.775	849.66	389.58	779.33	309.67	0.775
LF	809.00	791.02	966.67	1344.89	0.775	1045.71	473.39	802.83	408.15	0.475
HF	1041.29	1141.45	359.17	317.51	0.153	1164.86	602.69	536.17	527.43	0.037 *

BMI: body mass index, SDNN: Standard deviation of all NN intervals, rMSSD: square root of the mean of the sum of the squares of differences between adjacent NN intervals, AVG_IHR: average of the instantaneous heart rate (beats per minute), VLF: very low frequency power in ms^2^, LF: low frequency power in ms^2^, HF: high frequency power in ms^2^, <<: same values as baseline, ∗: difference is significant at level 0.05.
